# Spatial mode conversion of single photons at the C-band using in fiber long-period gratings

**DOI:** 10.1038/s41598-025-92394-x

**Published:** 2025-03-06

**Authors:** Rodrigo Amorim, Lars Grüner-Nielsen, Karsten Rottwitt

**Affiliations:** 1 Department of Electrical and Photonic Engineering, Technical University of Denmark, Ørsteds Plads 343, 2800 Kgs. Lyngby, Denmark; 2 Danish Optical Fiber Innovation, Åvendingen 22A, 2700 Brønshøj, Denmark

**Keywords:** Fibre optics and optical communications, Quantum optics, Single photons and quantum effects, Nonlinear optics

## Abstract

The ability to convert the spatial mode of single photons opens up a promising path to enhancing quantum communication protocols by enabling high-dimensional encoding and efficient multiplexing. In this work, we demonstrate spatial mode conversion of single photons at 1550.6 nm using a fiber long-period grating (LPG). The fundamental $\hbox {LP}_{01}$ mode was converted to higher-order modes $\hbox {LP}_{11}$ and $\hbox {LP}_{02}$, with quantum mode conversion efficiencies of 87.5 ± 1.4% and 96.1 ± 1.6%, respectively. The characterization of the converted single photons was carried out using a time-of-flight technique and coincidence measurements, by taking advantage of the differences in group velocity between the modes. We also performed loss measurements at the single-photon level and demonstrated mode re-conversion by using a second LPG to restore the photons back to the fundamental mode. These results highlight the potential of LPGs as a versatile tool for spatial mode manipulation at the single-photon level, with applications in high-dimensional quantum communication and nonlinear optical interactions.

## Introduction

The possibility of tailoring the spatial mode profile of single photons has been a hot topic of research for years now, due to the vast applications in the field of quantum information^1–3^. The ability to access different spatial modes provides an additional degree of freedom (DoF) that has emerged as a promising pathway for high-dimensional quantum key distribution (HD-QKD) and enhanced quantum communication capabilities^4^. Unlike the commonly used polarization DoF, which inherently forms a two-dimensional system where a photon can exist in a superposition of horizontal (H) and vertical (V) polarization states, the spatial mode DoF, in principle, offers infinite dimensions. In practice, this dimensionality is constrained by the number of modes that can be efficiently generated and controlled. Beyond the capacity advantages of high-order modes, they have also been demonstrated to exhibit greater resilience to noise compared to two-dimensional systems^5^. Another compelling application for single photons in high-order modes is their use in frequency conversion via nonlinear processes. In such processes, the wavelength of single photons can be tuned by interacting with a high-power pump laser inside a nonlinear material^6^. For efficient conversion, both energy and momentum conservation must be satisfied. A key strategy for achieving momentum conservation involves tailoring the spatial mode of the input photon to match the phase-matching conditions required for frequency conversion^7^. These applications highlight the need for devices that can efficiently manipulate the spatial mode of single photons.

A key element of these applications is the light source, which, to guarantee a secure quantum channel, requires it to be emitted in the single-photon regime^8^. Several techniques have been used to generate single photons based on solid-state materials (SSM)^9^, such as quantum dots^10^ and color centers in crystals^11^. Another widely used platform for generating single photons uses nonlinear phenomena such as spontaneous parametric down-conversion (SPDC) and spontaneous four-wave mixing (SFWM) in crystals^12^ and waveguides^13^, respectively. These processes simultaneously generate a pair of photons in which one of them can be used to herald the presence of the other.

Once the source is set, the next challenge is to engineer the spatial modes of single photons. There are two main approaches to this problem: (1) the photons are generated in the desired spatial mode. This has been demonstrated using SSM to generate free space orbital angular momentum modes^14^, and through the SFWM process in optical fibers^15,16^ and silicon-based waveguides^17,18^. (2) The photons are generated in a specific mode (usually in the fundamental mode) and are subsequently converted to the target mode using a passive device. In this case, spatial light modulators (SLM) are the most widely used converter, and their functionality has been extensively studied^19^. The drawbacks of SLMs are their bulkiness and the need for precise alignment. Photonic lanterns (PLs), in contrast, are fiber devices capable of converting light from a single-mode fiber (SMF) to different spatial modes propagating in a few-mode fiber (FMF)^20^ and have been qualitatively shown to work as a mode converter at the single-photon level^21^. In this study, we suggest the use of another fiber-based device called long-period grating^22^ (LPG) as an alternative to transform single photons from the fundamental linear polarized (LP) mode to higher-order $\hbox {LP}_{11}$ and $\hbox {LP}_{02}$ modes. LPGs are advantageous over PL devices due to their simpler production process and high conversion efficiency, which can reach 99%^23,24^. In contrast, PL devices are prepared for integration in space-division multiplexing (SDM) protocols^25^, simplifying their implementation in quantum communication networks.

Fiber-based LPGs were originally designed to couple the fundamental mode of single-mode fiber to cladding modes in order to create spectral filters i.e. notch filter^22^. Later, researchers explored their potential for generating higher-order propagating modes, such as LP modes, to compensate for dispersion in long fiber links^26^. More recently, LPGs started to be used for sensing considering that the light coupled to the cladding can interact with the surrounding medium^27,28^. To our knowledge, no previous attempts have been made to demonstrate the usability of LPG in the quantum regime, where a single-photon source replaces coherent light. In this work, we build upon our previous preliminary study^29^ by demonstrating the spatial mode conversion of single photons generated via the spontaneous four-wave mixing (SFWM) process in a hydrogenated amorphous silicon (a-Si:H) waveguide. Given that our experiments involve single photons, conventional methods for visualizing the spatial mode profile, to assess its content, require hardly available intensified charge coupled-device (ICCD) cameras^30^, therefore we used a time-of-flight technique with single-photon detectors to show the expected conversion. We also analyzed the insertion loss (IL) and mode conversion loss (MCL) of our device from the raw number of photons detected. Most applications involving single photons in high-order spatial modes require reconversion to the fundamental mode. This necessity arises primarily for two reasons. First, the majority of single-photon detectors operating in the communication band use single-mode fiber coupling, making it essential to reconvert the spatial mode for efficient detection. Second, applications such as HDQKD often employ a mode converter at the receiver side that selectively reconverts only a specific high-order mode back to the fundamental, leaving other modes unchanged^31^. This selectivity enables precise characterization of the transmitted spatial mode. To address these requirements, we demonstrate mode reconversion by implementing a second long-period grating (LPG) before the detectors. Bringing the LPG performance to the quantum regime allows us to experimentally characterize the quantum correlation of the photons after and before conversion through coincidence measurements, which is not possible in the classical regime. We thus hope to draw attention to the usability of LPGs as an extra tool in the toolbox for quantum protocols.

## Results

### Source characterization

**Fig. 1:**
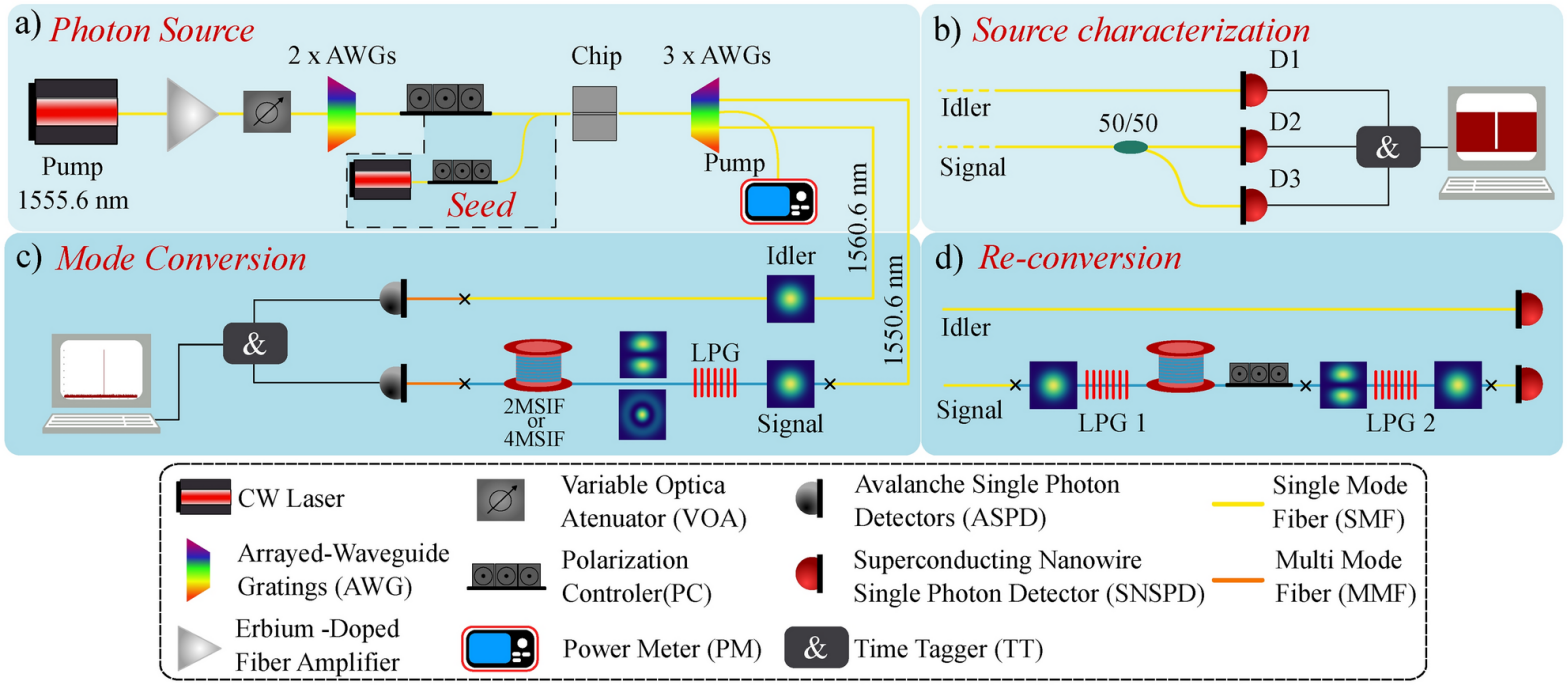
Schematic of the experimental setup. (**a**) Photon-pair generation through SFWM process in an a-Si:H chip pumped by a CW laser. The seed laser is only used to measure the spectrum of the process. (**b**) Setup used for $g^{(2)}$ measurement. (**c**) Mode conversion and detection part. The $\hbox {LP}_{11}$ conversion was achieved using the 2MSIF whereas the $\hbox {LP}_{02}$ with the 4MSIF, each conversion scheme have a LPG with different period. (**d**) Mode re-conversion setup.

**Fig. 2:**
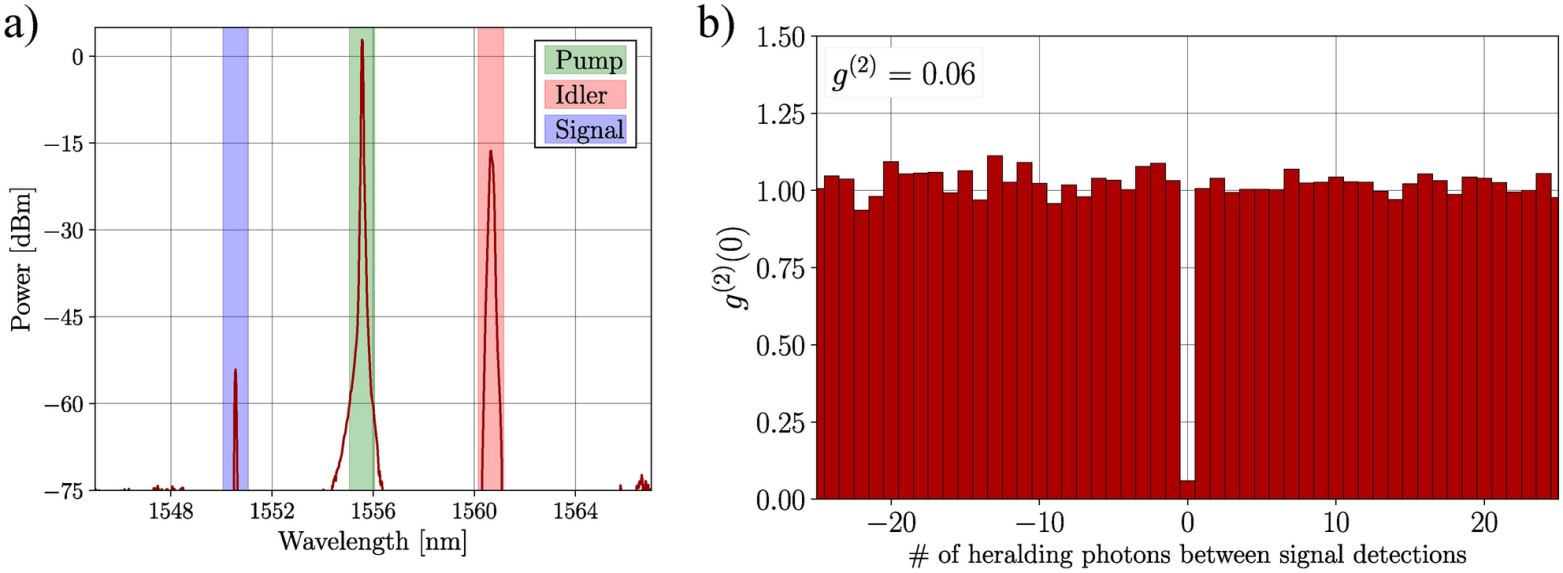
Four-wave mixing spectrum recorded by seeding the process with a CW laser at the idler wavelength (1560.6 nm). (**b**) Histogram obtained from the *g*^(2)^ setup. The dip at zero number of heralding photons between signal detections gives the value of *g*^(2)^, which is displayed in the top left of the histogram.

The first step of our LPG characterization is to generate single photons in the telecommunication band. To do that, we implemented the setup shown in Fig. 1a, which is based on an SFWM process triggered in an a-Si:H waveguide. A CW pump laser emitting at 1555.6 nm is amplified by an erbium-doped fiber amplifier (EDFA) and sent through a variable optical attenuator (VOA) and two stacked arrayed-waveguide gratings (AWG) to tune the pump power and filter out the pump sidebands amplified by the EDFA, respectively. A polarization controller (PC) is used to ensure the best possible light coupling into the waveguide. The waveguide is made of a rectangular a-Si:H core with transverse dimensions of 225 nm by 730 nm and a length of 3.3 mm surrounded by silica ($\hbox {SiO}_2$)^32,33^, and it has inverted tapers to facilitate in/out coupling using lensed fibers. The SFWM is achieved by the annihilation of two pump photons and the creation of a signal and an idler photon with higher and lower frequencies, respectively. A spectrum of the FWM process is shown in Fig. 2a. This was recorded by using a seed laser (inside the dashed box in the setup figure) at the idler wavelength, since the SFWM is not strong enough to show up in a typical optical spectrum analyzer (OSA). To effectively separate the signal from the idler and eliminate the strong pump field, we utilized three other AWGs, resulting in approximately 130 dB pump suppression. Using the AWG channel centered at the pump wavelength, we sent the pump to a power meter (PM) to estimate the coupled pump power in the chip. After filtering, the photon wavelengths were centered at 1550.6 nm for the signal and 1560.6 nm for the idler. To validate our claim of achieving mode conversion of single photons, we need to demonstrate that we are generating at most one pair of photons at a time. A common metric for characterizing the photon number statistics of a parametric source is the herald second-order autocorrelation function $g^{(2)}(\tau =0)$^34^. The experimental setup for measuring $g^{(2)}$ is illustrated in Fig. 1b. After the AWGs, the idler photons are directed straight to a superconducting nanowire single-photon detector (SNSPD) labeled D1. The signal photons, on the other hand, are split using a 50/50 fiber splitter and then sent to two SNSPDs, D2 and D3, in a Hanbury-Brown-Twists configuration^34^. All three detectors are connected to a time tagger, which allows the detection times to be recorded. Experimentally, $g^{(2)}$ is estimated using the following expression^35^:

1 $$\begin{aligned} g^{(2)}(0) = \frac{N_{123}N_1}{N_{12}N_{13}}, \end{aligned}$$

where $N_1$ represents the raw number of detections in the idler detector, also known as the herald arm. $N_{12(3)}$ refers to the number of coincidental detections between the herald photon and the photons reaching detector D2 (D3), and $N_{123}$ is the number of threefold coincidences, i.e., simultaneous coincidences between D1, D2 and D3. The value of $g^{(2)}$ characterizes the photon number statistics of the source. If $g^{(2)} < 1$, the source has nonclassical statistics, e.g., sub-Poissonian, and if its value is less than 0.5, the source emits single photons^13^. It is important to note that a perfect single photon emitter has $g^{(2)} = 0$. This can be seen by inspecting equation (1) and noticing that if just one pair is generated at a time and there are no noise photons, threefold coincidences never occur. Because we are pumping with a CW laser, our source is called asynchronous^36^, which means that we cannot build the commonly adopted $g^{(2)}$ versus time delay histogram^37^. Instead, we show in Fig. 2b a similar histogram where we used the number of herald detections (Idler photons) between two consecutive signal detections recorded in different detectors^38^. The dip at zero reflects the antibunching behavior of the single-photon source. The $g^{(2)}$ extracted through the histogram (shown as an inset in Fig. 2b) matches the number obtained using Eq. (1). The value of 0.06 for the $g^{(2)}$ function shows that our source is emitting light in the single photon regime, i.e., at most one pair at a time. The $g^{(2)}$ depends on the pump power; therefore, all the following measurements were taken using the same pump power used for the $g^{(2)}$ measurement, which is approximately 3.5 mW (coupled into the waveguide). More information on the quantum performance of the chip can be found in references^32,39^.

### Mode conversion scheme

**Fig. 3:**
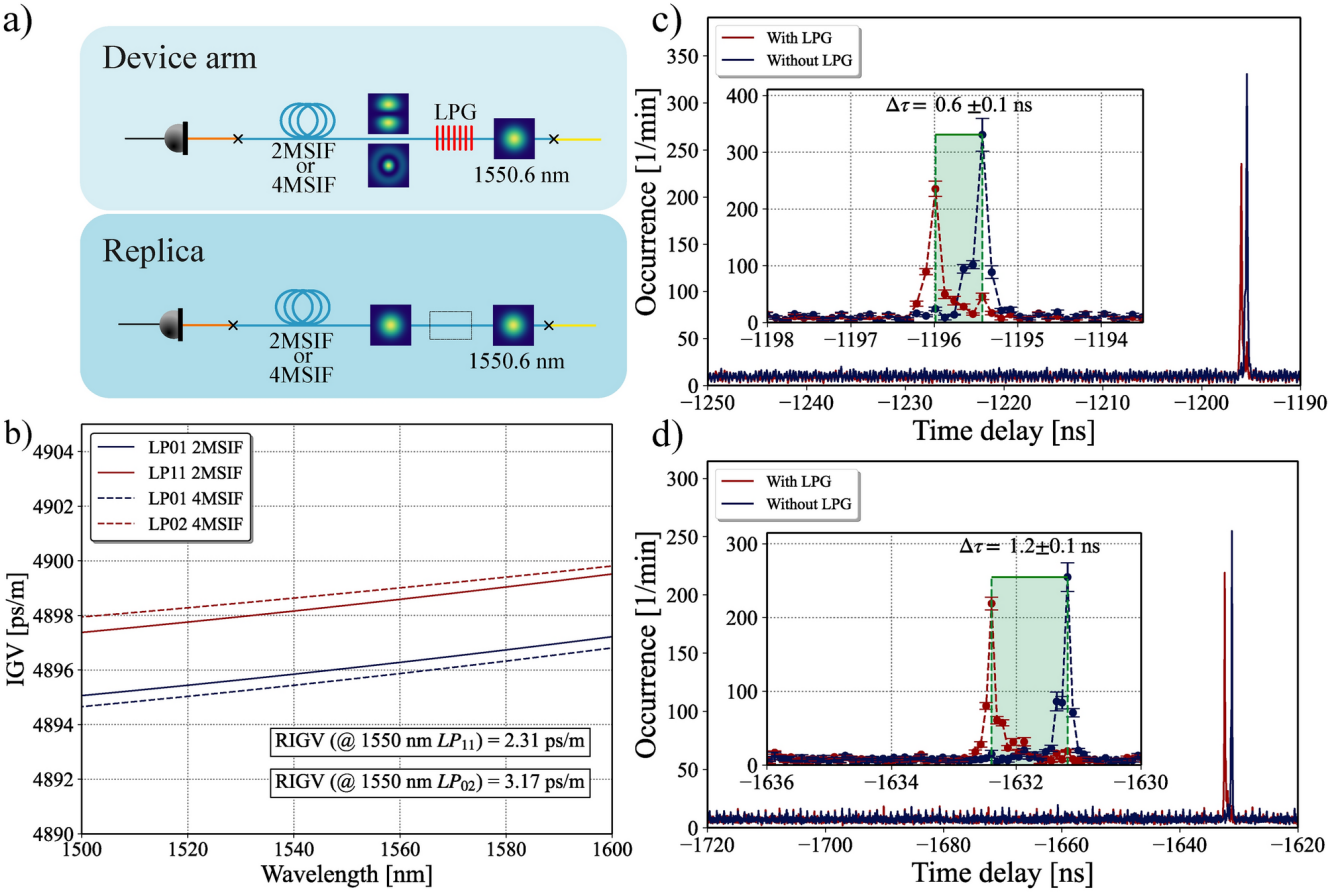
(**a**) Sketch to illustrate the procedure used to compare the time-of-flight of the converted (Device arm) and nonconverted (Replica arm) signal photons. (**b**) Simulated Inverse group velocity of the involved spatial modes in the 2MSIF and 4MSIF. (**c**) and (**d**) show the coincidental histogram versus time delay between the signal and idler photons for the LP_11_ and LP_02_ conversion, respectively. The two traces in each histogram were obtained with the device arm (with LPG) and replica arm (without LPG). The error bars represent one standard deviation from the mean number of coincidences for each time bin.

Now that we have characterized the photon statistics of our source, we continue with the mode conversion scheme. The setup is shown in Fig. 1c. After splitting the signal and idler using the AWGs, we send the idler directly to a multimode avalanche photon detector (ASPD). The signal photons are coupled via a splice to either a 260 meter two-mode step index fiber (2MSIF) or a 350 meter four-mode step index fiber (4MSIF)^40^, each with an engraved LPG designed to convert the linearly polarized (LP) mode $\hbox {LP}_{01}$ to the $\hbox {LP}_{11}$ or $\hbox {LP}_{02}$ mode, respectively (see Supplementary Material for more information about the fibers). In this study, the LPG was fabricated by positioning the fiber near a thin, heated platinum wire^41^. The refractive index of the fiber changes locally due to heat, and the grating is formed by periodically moving the wire along the fiber. The distance between the heated points gives the grating period $\Lambda$. Using this technique, once the grating is inscribed in the fiber, it cannot be tuned. As a result, it functions similarly to a waveplate, requiring a dedicated LPG for each specific mode of interest. To enable the coupling of light to the higher-order mode, the grating period must satisfy the phase-matching condition^42^:

2 $$\begin{aligned} \Lambda = \frac{\lambda }{n_{eff}^{01}-n_{eff}^{lm}}, \end{aligned}$$

where $\lambda$ is the target wavelength of the mode conversion and $n_{eff}^{01}$ and $n_{eff}^{lm}$ are the effective refractive indices of the fundamental mode and the $\hbox {LP}_{lm}$ mode of the fiber, respectively, at the target wavelength. Although Eq. (2) provides the grating period for a specific wavelength, LPGs inherently operate over a conversion bandwidth. If the bandwidth of the single-photon source exceeds that of the LPG, it may affect the conversion efficiency. However, this is not a concern in our setup, as the photon bandwidth is limited by spectral filters with a 200 GHz bandwidth, which is significantly narrower than the tens of THz bandwidth supported by the LPG (see Supplementary Material). By simulating the fibers, we found the expected effective refractive index for all propagating modes of the fibers, giving a period of approximately 1190 μm for the $\hbox {LP}_{11}$ conversion and 607 μm for the $\hbox {LP}_{02}$ conversion. A total of 9 and 31 periods were written on the fibers for the $\hbox {LP}_{11}$ and $\hbox {LP}_{02}$ conversions, respectively, which made the devices compact, i.e., around 1.5 cm. Using coherent light, we achieved up to 99% conversion efficiency and showed low pump polarization dependency on the mode conversion (see Supplementary Material). Since LPGs are fiber-based passive devices, they are inherently stable and do not exhibit instabilities due to mechanical drifting or misalignment when kept fixed. The aforementioned efficiency was achieved by conducting traditional measurements such as spectrally and spatially resolved imaging ($\hbox {S}^2$)^43^ using a camera and analyzing the transmission spectrum using an OSA. These measurements would require rarely available equipment such as ICCD to work on single-photon sources^21^; therefore, we employed a time-of-flight technique to show the mode conversion. The measurement procedure works as follows: First, we send the signal photons through the LPG written in the 2MSIF or 4MSIF, depending on the mode we want to characterize. Then, the signal and idler are collected in the ASPD, generating a coincidence histogram. Next, we keep the idler arm unchanged while substituting the signal arm with an identical replica. Everything is exactly as before, except we remove the LPG and replace it with a section of 2MSIF or 4MSIF that corresponds to the length of the LPG. Consequently, the signal photons propagate in the $\hbox {LP}_{01}$ mode throughout the entire fiber length. As previously, we record the coincidence histogram of this replica setup. Figure 3a shows a schematic of this procedure. Because the source generates both the signal and idler at the same time, we expect to see a peak of coincidences at a delay time corresponding to the optical length difference between their arms. Importantly, since different fiber modes have different propagation velocities, we expect to be able to observe a shift in the delay time of the coincidence histogram when we compare the measurements with and without the LPG. This shift depends on the group velocity difference between $\hbox {LP}_{01}$ and the converted modes, i.e., $\hbox {LP}_{11}$ for 2MSIF and $\hbox {LP}_{02}$ for 4MSIF. In Fig. 3b, we show the simulated inverse group velocity of the involved modes in which the relative inverse group velocity (RIGV) between the $\hbox {LP}_{01}$ and the $\hbox {LP}_{11(02)}$ is 2.31 ps/m (3.17 ps/m). From the simulations, we expect to see a peak shift of approximately 0.6 ns between the replica and device setup for the $\hbox {LP}_{11}$ conversion scheme. Figure 3c shows the experimental results. The blue trace is the histogram of the replica setup without the LPG, and the red trace is the histogram of the setup with the LPG. We see that the photons propagating through the LPG take approximately 0.6 ns more to reach the detectors than those propagating through the replica, agreeing with the simulations. In Fig. 3d, we show the results for the $\hbox {LP}_{02}$ conversion. From the simulations, we expect to see a delay difference between the replica and the device arm of 1.1 ns, which agrees with the value obtained experimentally.

A common metric for evaluating single-photon generation is the coincidence-to-accidental ratio (CAR), which characterizes the noise level of a single-photon source^44^. The CAR may be extracted from our coincidental histogram, since the CAR is defined as $CAR = \frac{N_{coin}-N_{acc}}{N_{acc}}$, where $N_{coin}$ is the number of coincidental detections and $N_{acc}$ is the number of accidental coincidence detections. Coincidental detections correspond to the peak in the histogram, once the signal and idler are generated simultaneously. Conversely, accidental counts arise from noise photons resulting from Raman scattering within fibers and the waveguide, pump leakage, and the dark counts of detectors. These noise photons in the signal and idler arms lack time correlation, meaning that they are not produced simultaneously, causing their coincidences to populate all time bins in the histogram. For single-photon source applications such as QKD, a CAR exceeding 10 is necessary^45^. From the histogram in Fig. 3c, we measured a CAR of 32 and 28 for the replica and device arms, respectively. For the histogram in Fig. 3d, the recorded CAR is 34 for the replica and 30 using the LPG. By examining the histograms, one can observe that the peaks exhibit asymmetry and small kinks on their sides. We believe these features have no physical significance and are instead a result of the time jitter of the detectors, which is approximately 100 ps. Additionally, the observed asymmetry varies with the chosen bin width and its central positioning.

In Fig. 3c we see a small peak in the histogram with the LPG at the same time delay where the peak without the LPG is located. This shows that not all photons were converted to the $\hbox {LP}_{11}$ mode by the LPG. Therefore, we can define a quantum conversion efficiency as:

3 $$\begin{aligned} \eta _i = \frac{N_{coin}^{i} - N_{acc}}{N_{coin}^{i}+ N_{coin}^{01}-2N_{acc}}, \end{aligned}$$

where $N_{coin}^{01}$ is the sum of coincidence counts within the peak corresponding to the unconverted $\hbox {LP}_{01}$ photons, $N_{acc}$ is the average number of accidental coincidences (i.e., background noise), and $N_{coin}^{i}$ is the sum of coincidence counts corresponding to the converted photons in mode $\hbox {LP}_{i}$, with $i = 11 \text { or } 02$. For the $\hbox {LP}_{11}$ mode, we measured a conversion efficiency of $87.5 \pm 1.4\%$. In Fig. 3d, no peak corresponding to photons in the $\hbox {LP}_{01}$ mode is observed when the LPG is used. This suggests a significantly higher conversion efficiency for the $\hbox {LP}_{02}$ mode. Using Eq. (3), we estimate a conversion efficiency of $96.1 \pm 1.6 \%$. The Supplementary Material includes data on the classical conversion efficiency for the LPGs used in this study, where both LPG types demonstrate a conversion efficiency of 99%. We attribute the difference in conversion efficiency, in the $\hbox {LP}_{11}$ case, to mode coupling at the splice point between the LPG and the 260-meter spool of 2MSIF. We refer to $\eta _i$ as the quantum conversion efficiency because it can only be determined by measuring coincidence counts, a measurement exclusively possible in the quantum regime.

### Loss analysis

The applicability of LPGs for single-photon conversion also depends on the insertion loss (IL) and mode conversion loss (MCL) provided by the device. We estimate the IL of the LPGs from the mode conversion setup of Fig. 1c, where instead of looking at the coincidences, we check the total number of counts in the signal and idler detectors. Table 1 shows the detection rates for the aforementioned histograms. The uncertainties in the count rates correspond to one standard deviation of the mean count rate, measured over a five-minute interval for each scenario (i.e., replica and device). We define the mode conversion loss as the ratio of signal photon counts detected with and without the LPG. However, a direct comparison of these two measurements can be misleading, as fluctuations in input power between the two setups, i.e., the replica and the device setup shown in Fig. 3a, may slightly affect the number of generated photon pairs. To account for these variations, we correct the loss measurement by referencing the count rate in the untouched idler arm. Thus, the mode conversion loss is estimated using the following expression

4 $$\begin{aligned} MCL = 10\log \left( \dfrac{N_{Signal}^{D}}{N_{Signal}^{R}\times \dfrac{N_{Idler}^{D}}{N_{Idler}^{R}}}\right) , \end{aligned}$$

where $N_{i}^{D(R)}$ is the number of counts in arm *i* ($i = Signal, Idler$) using the Device (Replica) setup. The obtained MCLs are lower than 0.82 dB and 0.17 dB for the $\hbox {LP}_{11}$ and $\hbox {LP}_{02}$ conversion schemes, respectively. Even though the number of idler counts is always greater than the number of signal counts due to Raman noise, we can estimate the upper boundary IL of the LPG setup, i.e., considering splicing loss, MCL and propagation loss, by the ratio $N_{Signal}^D/N_{idler}^D$. In this manner, we estimate ILs of less than 1.42 dB and 0.88 dB for the $\hbox {LP}_{11}$ and $\hbox {LP}_{02}$ conversions, respectively.

**Table 1:** List of count ratios measured in the signal and idler detectors with the Replica and Device scheme along with the respective mode coupling loss (MCL) and insertion loss (IL).

LPG type	Setup	$\hbox {N}_{Signal}$ [Hz]	$\hbox {N}_{Idler}$ [Hz]	MCL [dB]	IL [dB]
${LP}_{11}$	Replica	${36757 \pm 27}$	${42306 \pm 19}$	$< 0.82$	$< 1.42$
	Device	${30588 \pm 56}$	${42246 \pm 18}$		
${LP}_{02}$	Replica	${35054 \pm 86}$	${41511 \pm 72}$	$< 0.17$	$< 0.88$
	Device	${34619 \pm 09}$	${42313 \pm 09}$		

### Re-conversion

**Fig. 4:**
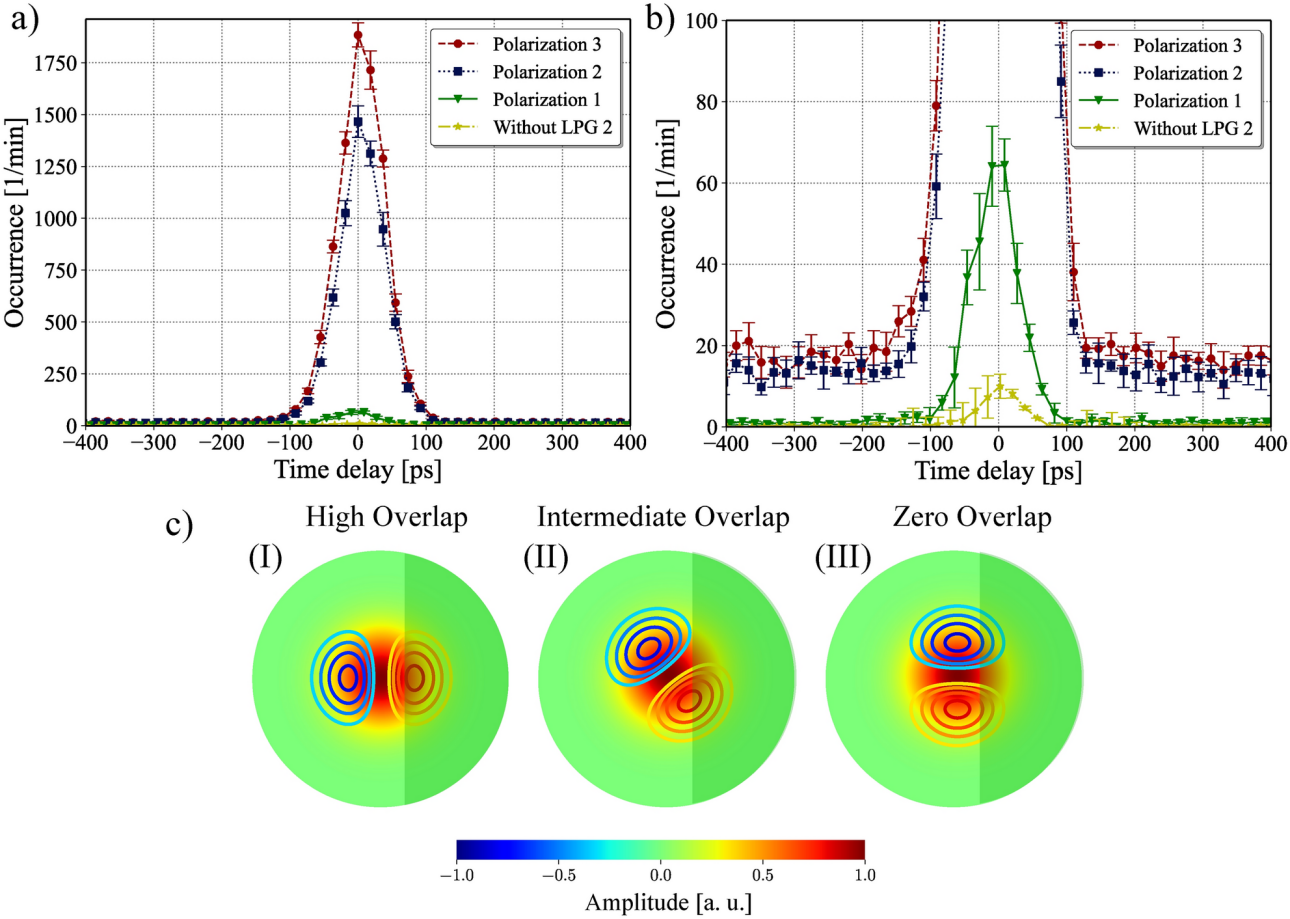
(**a**) Coincidence histogram obtained using the re-conversion setup for different positions of the polarization controller. The star trace was measured by splicing the end of the 2MSIF to a SMF without the LPG 2. (**b**) Zoomed version of the first plot showing that almost no re-conversion can be achieved by changing the polarization. (**c**) Illustration showing the importance of the LP_11_ mode profile orientation for the coupling coefficient.

For some applications, it might be important to reconvert the photons in the high-order modes to the fundamental mode to operate in a standard single-mode fiber network. To show the possibility of re-conversion, we used the setup shown in Fig. 1d. LPG 1 converts the signal photons from $\hbox {LP}_{01}$ to $\hbox {LP}_{11}$. Then, the converted photons travel through the 260 meter 2MSIF spool, where LPG 2 is spliced at its end. The LPG 2 is also designed to convert the fundamental mode to the $\hbox {LP}_{11}$, and since the gratings are reciprocals, it also works as a reconverter. The end of the second LPG is spliced into an SMF pigtail and detected using an SNSPD. In between the two LPGs, we inserted a PC to rotate the spatial profile of the photons. The idler photons are sent directly to another SNSPD. In Fig. 4a we show the coincidence histogram obtained in the re-conversion setup. The coincidence peak was centered at 0 delay time in the post-processing analysis to facilitate visualization. The different traces labeled Polarization 1, 2, and 3 represent different positions of the PC. The star marker trace, visible in the zoomed-in version of the histogram in Fig. 4b, represents the coincidences measured after removing LPG 2 and splicing the 2MSIF spool directly to the SMF, i.e., without reconverting the high-order mode to the fundamental mode. The re-conversion process is significantly influenced by polarization. This can be explained by the fact that when creating the LPG, we selectively heated only one side of the fiber. As a result, the refractive index perturbation is limited to that specific side of the fiber. According to mode coupling theory, the efficiency of mode conversion is directly proportional to the overlap integral between the two involved modes i.e. $\hbox {LP}_{01}$ and $\hbox {LP}_{11}$ and the perturbation profile. The coupling coefficient is^46^

5 $$\begin{aligned} \kappa _{01-11} \propto \int _{0}^{2\pi }\int _{0}^{\infty } E_{01}(r,\phi )E_{11}^*(r,\phi )f(r, \phi )\, r \, dr \, d\phi , \end{aligned}$$

where $E_{01(11)}$ is the electrical field amplitude of the $\hbox {LP}_{01(11)}$ mode, $f(r,\phi )$ is the index perturbation profile function and the integration is taken over the transverse profile of the fiber. Figure 4c shows the spatial mode profiles inside the fiber with the perturbation (darker region) and illustrates the dependence of the mode profile orientation to achieve re-conversion. For case $\left( \text {I}\right)$, the modes overlap maximally with the perturbation. By changing the PC, we rotate the mode profile and an orientation similar to that in case $\left( \text {II}\right)$ can be achieved, which has a smaller overlap. Continually adjusting the PC, the mode can be oriented as in case $\left( \text {III}\right)$, resulting in a zero overlap integral. It is important to realize that this analysis works for the re-conversion scheme, where the incoming photons are in the asymmetric mode. For the first conversion, i.e., in LPG 1, since the incoming photons are in the fundamental mode, the mode coupling is independent of the orientation of the polarization.

## Discussion

We achieved spatial mode conversion of single photons in the C-band using long-period gratings (LPGs). Specifically, we converted the fundamental modes of a two-mode step-index fiber and a four-mode step-index fiber to the $\hbox {LP}_{11}$ and $\hbox {LP}_{02}$ modes, respectively. This demonstrates the usability of LPGs to convert both symmetric and asymmetric modes effectively. To characterize the mode conversion, we implemented a time-of-flight analysis, which proved to be an effective method for distinguishing single photons in a high-order spatial mode. We showed that after conversion, the coincidental-to-accidental ratio was slightly lower (approximately by 12%), which means that LPGs could also be used in QKD protocols. Through coincidence measurements, we determined the quantum mode conversion efficiencies to be $87.5 \pm 1.4\%$ for the $\hbox {LP}_{11}$ mode and $96.1 \pm 1.6 \%$ for the $\hbox {LP}_{02}$ mode. The insertion loss of our devices was measured to be at most 1.42 and 0.88 dB for the $\hbox {LP}_{11}$ and $\hbox {LP}_{02}$ modes, respectively. Given that part of these losses occurs at the splice points, we estimate the mode conversion loss to be 0.82 and 0.17 dB, respectively, which is comparable to that of other devices such as PL^21^. We also demonstrate mode re-conversion where we placed a second LPG to convert the high-order mode $\hbox {LP}_{11}$ back to the fundamental mode and the importance of the orientation of the mode profile for achieving efficient re-conversion.

In conclusion, we hope to draw attention to the potential of using LPGs as an effective means of achieving mode conversion of single photons for quantum protocols. It is noteworthy that the entire setup, post single-photon generation, is fiber-based, which mitigates extra losses and alignment issues that may arise from free-space coupling. Additionally, LPGs are also simpler to operate and more affordable compared to other mode converters such as spatial light modulators and photonic lanterns. However, it is important to note that LPGs have a relatively narrowband and low tunability, as each LPG is designed for a specific type of mode conversion, necessitating the creation of multiple devices for different modes and wavelengths.

Our demonstration of mode conversion at the single-photon level paves the way for the implementation of LPGs in quantum communication protocols, particularly high-dimensional quantum key distribution (HD-QKD), where the use of high-order modes can significantly enhance information capacity. While QKD is a promising avenue, we believe that the most impactful application of our work lies in frequency conversion using nonlinear interactions in optical fibers. We emphasize that beyond these specific applications, any scenario requiring single photons in high-order modes can benefit from the use of LPGs due to their high conversion efficiency, low loss, and, as demonstrated in our study, their ability to preserve photon correlations. These features position LPGs as versatile and reliable components for scalable quantum technologies.

## Methods

We used a CW laser (Photonetics TUNICS-BT, 400 kHz linewidth) to pump the SFWM and a similar laser (ANDO AQ4321D) as the seed to acquire the spectrum shown in Fig. 2. The AWGs used have a bandwidth of 200 GHz, where we collected light from channel 21, 27 and 33 for the idelr, pump and signal, respectively. For the mode conversion setup, we used two ASPDs (IDQ 230 NIR) with approximately 25% quantum efficiency, 150 ps resolution, and 50 μs dead time. Connected to these detectors, we used a time tagger (*qutools quTAG*) with 81 ps resolution. For the source characterization and mode re-conversion setups, we used the time tagger (*qutools quTAG HR*) with 1 ps resolution and SNSPDs from *Quantum Opus* with a quantum efficiency of approximately 83% and 50 ns dead time. We opted for APDs over SNSPDs in the conversion measurements as they allowed for the connection of a multimode fiber.

## Supplementary Information

Supplementary Information.

## Data Availability

The data generated and/or analyzed in this study are available from the corresponding author on reasonable request.
