# Supplementary material for: Spatial mode conversion of single photons at the C-band using in fiber long-period gratings
Source: Sci Rep. 2025 Mar 6;15:7795. doi: 10.1038/s41598-025-92394-x (PMC11883002; doi:10.1038/s41598-025-92394-x)
Supplement: Supplementary file 1 — Supplementary Information. [file 41598_2025_92394_MOESM1_ESM.pdf]

Supplementary Information

Rodrigo Amorim[1,*], Lars Grüner-Nielsen[1,2], and Karsten Rottwitt[1]

[1]Department of Electrical and Photonic Engineering, Technical University of Denmark, Ørsteds Plads 343, 2800 Kgs. Lyngby, Denmark
[2]Danish Optical Fiber Innovation, Avendingen 22A, 2700 Brønshøj, Denmark
[*]rodsi@dtu.dk

Fiber and Long period grating details

The long-period gratings (LPGs) used in this work were made in-house using a technique described in detail in reference[1]. Therefore, in this section, we will focus on describing the fibers where the LPGs were written and also some relevant features of the LPGs devices itself. The classical characterization of the LPGs was obtained by performing spatial and spectral imaging (S^2) measurements and analyzing the transmitted spectrum of the light.

Two different step-index fibers were used to produce the LPGs. For LPGs that convert the fundamental mode of the fiber into the LP_{11} mode, we used the *OFS* two-mode step-index fiber (2MSIF) with a core and cladding diameters of 19 μm and 125 μm, respectively, and a refractive index contrast of $\Delta n = 4.8 \times 10^{-3}$ at 1550 nm. For the LPG designed to convert the LP_{01} fiber mode to the LP_{02} mode, we used a *OFC* Four-mode step-index fiber (4MSIF), which has a similar index profile but a larger core diameter of 25 μm. The names 2MSIF and 4MSIF is related to the number of modes guided by the fibers at the C-band wavelengths. This is also the reason why the 4MSIF had to be used for the LP_{02} conversion, once this mode is not guided in the 2MSIF. In Fig. S1, we show the simulated effective refractive index difference (Δn_{eff}) between the refractive index of the cladding and the effective refractive index of the modes as a function of wavelength. The simulated values are found by solving the scalar wave equation using the finite difference method. The red strip indicates the cutoff wavelength for each mode. Modes with Δn_{eff} smaller than the refractive index of the cladding do not propagate. In the inset, we see that for 1550 nm the mode LP_{02} should be a propagating mode of the 2MSIF but, since it is too close to the cutoff point, any small bend would be enough to couple the mode to the cladding, and therefore, in practice, only the LP_{01} and LP_{11} are guided in that fiber.

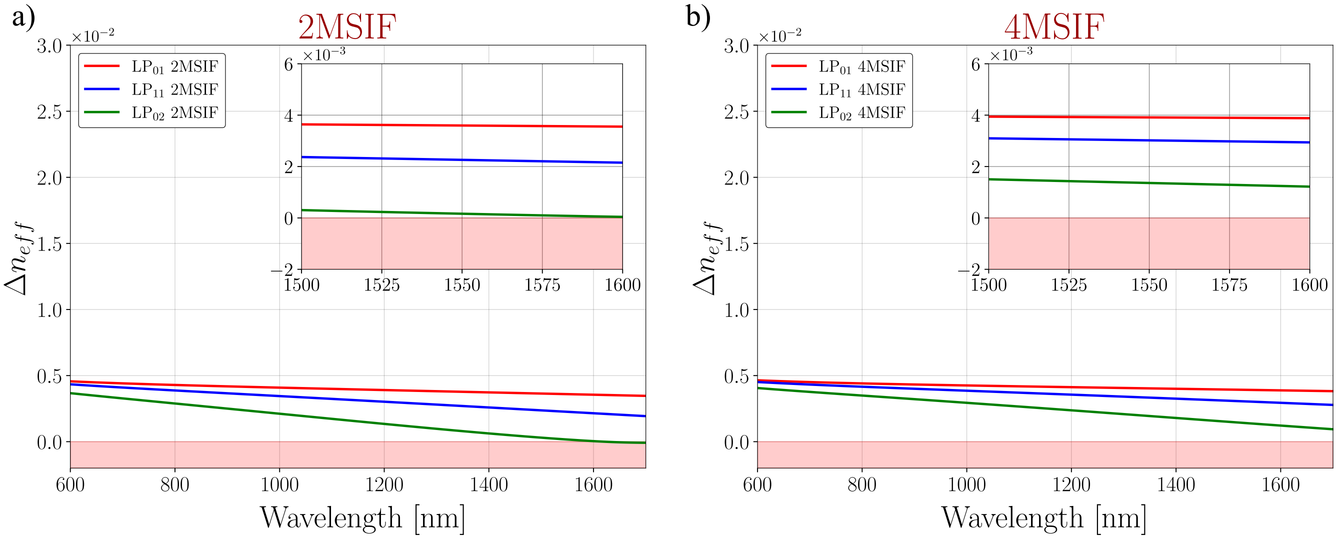

Figure S1. Simulation of the refractive index contrast versus wavelength for the three modes of interest in both 2MSIF and 4MSIF.

The LPG mode converter consists of a splice from the input single-mode fiber (SMF) to the few-mode fiber, followed by a

mode stripper and the LPG itself. The mode stripper ensures that only the fundamental mode is launched into/received from the LPG. The mode stripper consists of two orthogonal bends of 16 mm radius applied to the fiber. The LPG mode converters were classically characterized using two techniques. The first consists of analyzing the transmission spectrum of the device, and the second is by performing a spatially and spectrally resolved imaging measurement (S^2)[2]. We measured the transmission by passing a broad-band light source through the mode converter device, with a second mode stripper placed at the output. An SMF pigtail was spliced after the mode stripper and connected to an optical spectrum analyzer (OSA) for analysis. In Fig. S2a, we present the transmission spectra for two LPGs designed to convert the LP_{01} mode to the LP_{11} mode, which were used in the conversion and reconversion experiments discussed in the main text. Additionally, we show the spectrum for the LPG used to convert the LP_{01} mode to the LP_{02} mode. The dip observed in the spectrum results from the light being converted to higher-order modes, which are subsequently removed by the combination of the mode stripper and the spliced SMF. For all LPGs, the transmission loss is more than 20 dB at 1550 nm, indicating a mode conversion efficiency of more than 99%.

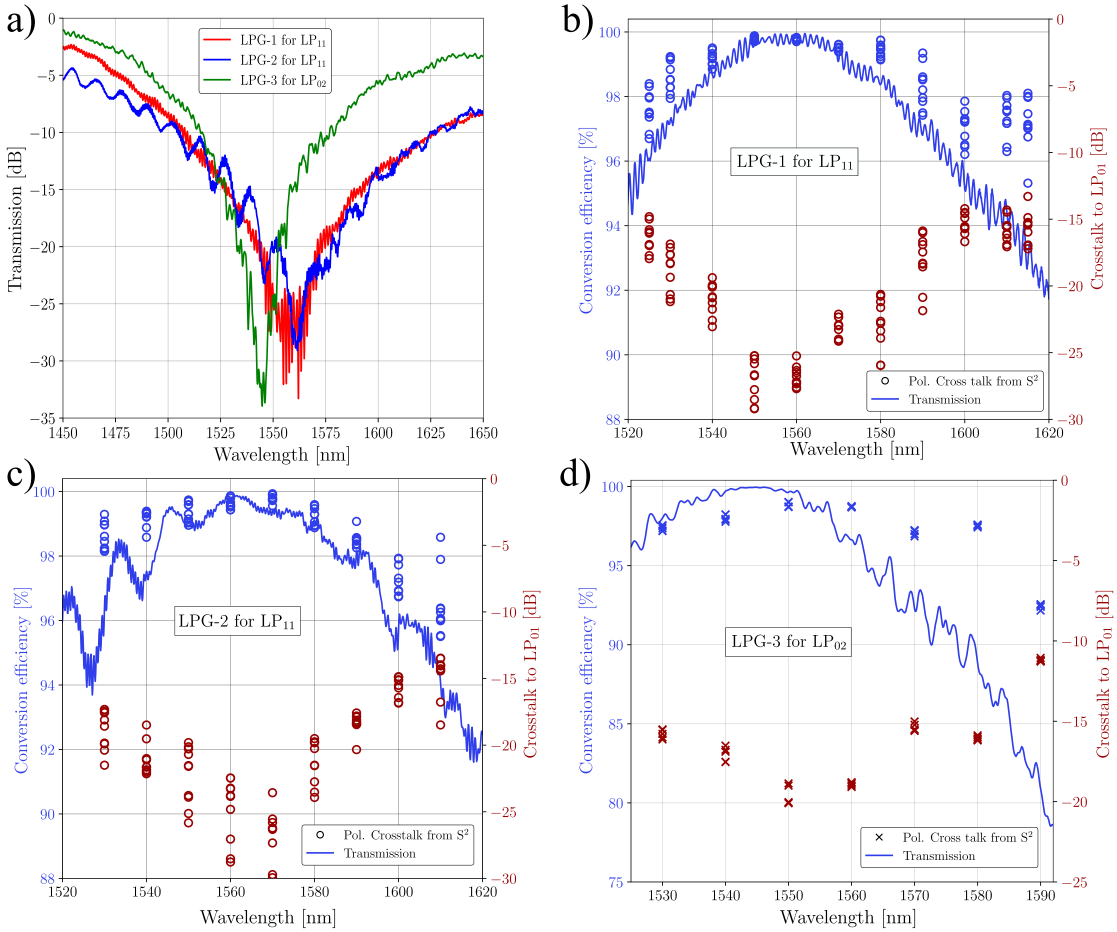

Figure S2. Classical characterization of the LPGs used for the single-photon conversion. **a)** Transmission spectrum of the LPGs. Conversion efficiency and mode crosstalk obtained by performing an S^2 measurement **b)** for the LPG-1 that converts the LP_{01} to LP_{11}, **c)** the LPG-2 that perform the same conversion and **d)** the LPG-3 that converts the LP_{01} into LP_{02}.

In Fig. S2b-d we show the results of the S^2 measurement. The procedure for measuring S^2 is described in reference[3]. S^2 measurements with ten arbitrary different input polarizations are taken for each wavelength. The scatter plot represents the estimated conversion efficiency and the crosstalk between the expected high-order mode, i.e., LP_{11} or LP_{02}, and the fundamental mode. The line traces are the conversion efficiency estimated from the transmission measurement. We can observe that all the LPGs have more than 30 nm bandwidth where the conversion efficiency is higher than 95 % and low polarization dependence. Also, we see that for the first two LPGs the transmission and the S^2 measurements agree with each other, but the LPG designed to convert to the LP_{02} mode does not. We suspect that this discrepancy arises due to the coupling of light into modes other than LP_{02}, such as LP_{11} and LP_{21}, as these modes are also guided in the 4MSIF, as shown in Fig. S1.

References

1. Akrami, P., Grüner-Nielsen, L., Rishøj, L. S. & Rottwitt, K. Fabrication of heat-induced long-period gratings for mode conversion in few-mode fibers. In Li, G. & Nakajima, K. (eds.) *Next-Generation Optical Communication: Components, Sub-Systems, and Systems X*, vol. 11713, 4, DOI: 10.1117/12.2576686 (2021).

2. Nicholson, J. W., Yablon, A. D., Fini, J. M. & Mermelstein, M. D. Measuring the modal content of large-mode-area fibers. *IEEE J. Sel. Top. Quantum Electron.* **15**, 61–70, DOI: 10.1109/JSTQE.2008.2010239 (2009).

3. Grüner-Nielsen, L., Mathew, N. M. & Rottwitt, K. Invited paper: Characterization of few mode fibers and devices. *Opt. Fiber Technol.* **52**, 101972, DOI: 10.1016/j.yofte.2019.101972 (2019).
